# Supplementary material for: Establishment of Human Leukocyte Antigen-Mismatched Immune Responses after Transplantation of Human Liver Bud in Humanized Mouse Models
Source: Cells. 2021 Feb 23;10(2):476. doi: 10.3390/cells10020476 (PMC7927063; doi:10.3390/cells10020476)
Supplement: Supplementary file 1 [file cells-10-00476-s001.pdf]

Supplemental Figure 1.

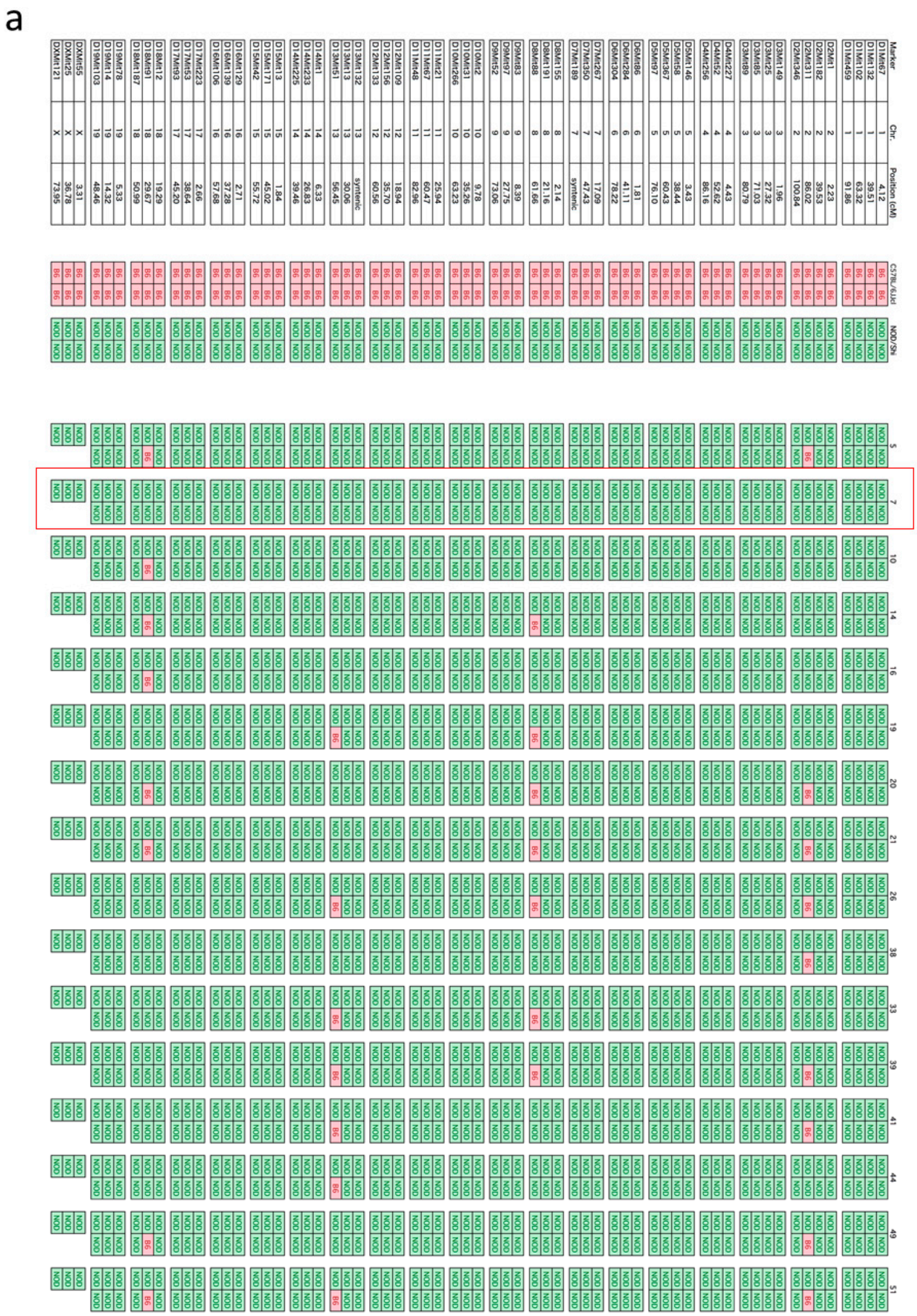

Supplemental Figure S1. NOG-HLA-A2Tg mice microsatellite screening. No7 strain was replacement of the genetic background from B6 to NOD was confirmed.
